# Comparison of intra operative hemorrhage by blunt and sharp expansion of uterine incision at cesarean section

**DOI:** 10.12669/pjms.37.7.4159

**Published:** 2021

**Authors:** Faiza, Farhadia Sadaf, Behzar Ameena, Nadia Rashid Khan

**Affiliations:** 1 Dr. Faiza, FCPS. Senior Registrar, Department of Obstetrics and Gynecology, Saidu Teaching Hospital, Swat, KPK, Pakistan; 2 Dr. Farhadia Sadaf, FCPS. Associate Professor, Department of Obstetrics and Gynecology, Saidu Teaching Hospital, Swat, KPK, Pakistan; 3 Dr. Behzar Ameena, FCPS. Specialist Gynecologist Rafique Shaheed Trust Hospital, Faisalabad, Pakistan; 4 Dr. Nadia Rashid Khan, FCPS. District Gynecologist, Category D Hospital Katlang, Mardan, Pakistan

**Keywords:** Caesarean section, Blunt and sharp expansion of uterine incision, Fall in HB and HCT

## Abstract

**Objectives:**

To compare the effect of blunt and sharp incision of uterus at cesarean section on intra-operative haemorrhage.

**Methods:**

This trial was conducted at the Department of Obstetrics and Gynaecology, Pakistan Ordinance Factory Hospital, Wah Cantt from 14^th^ January to 13^th^ July 2012. Total 80 women planned for lower segment cesarean section through Pfannensteil incision were randomized to either blunt uterine incision (Group-A, n=40) or sharp uterine incision (Group-B, n=40). The fall in Haemoglobin and HCT was compared in two groups and analyzed with help of SPSS version 10.

**Results:**

Both groups were similar in terms of demographic features like age, parity, gestational age and indication for cesarean section. The participants in Group-A reveled significantly less drop of mean Hb concentration as compared to Group-B (1.47±1.08 and 1.95±0.85 respectively, P value 0.031). Similarly, the fall in mean HCT was significantly less in Group-A in comparison to Group-B (3.21±1.3 and 4.21±2.17 respectively, P-value 0.015)

**Conclusion:**

Blunt expansion of uterine incision during caesarean section is associated with less fall in Haemoglobin and HCT as compared to sharp expansion.

## INTRODUCTION

Cesarean delivery is an operating procedure used to deliver babies that is indicated to avoid maternal and neonatal mortality. However, it is associated with short- and long-term hazards. CS rates have increased, and efforts are being made to ensure that CS is performed only when essential.^1^ Rates of cesarean delivery have augmented by about 7.0% to 31% in recent years.^2^ Varieties of surgical techniques are there to adopt, but very little data is available about proper technique to inform and to adopt. As the rates of postoperative morbidity due to cesarean delivery are higher in developing countries, improvements in health from proper cesarean delivery technique are likely to be significant in these countries.^3^

Safety of both mother and baby are essential during any type of delivery. The risk to health of mother is more during cesarean delivery as compared to vaginal birth and this risk accentuates as number increases.^4^

The possibility for major intraoperative blood loss remains an important apprehension for surgeons and anaesthetists.^5^ There are several surgical techniques of cesarean section which affect blood loss during operation such as spontaneous versus manual extraction of placenta, exteriorization or intra-abdominal uterine incision repair and one that is less debated is type of uterine incision blunt or sharp.^6^ Different surgeons adopt different techniques according to clinical situation and their own preferences,^7^ but it is important to adopt a surgical technique that will reduce blood loss during operation.^8^ At cesarean section, expansion of uterine incision is done by either cutting the myometrium sharply with scissors laterally and cephalad or bluntly by tearing with fingers.^6^ Some studies have reported that both techniques of uterine incision equally affect blood loss.^7^ Proponents of blunt dissection favors it because of decreased blood loss.^7^ The main drawback of blunt expansion of uterine incision is unintended tears into broad ligament, vagina or cervix, a main factor of arrest disorder^7^ or extension of incision into uterine blood vessel if fingers of surgeon swept laterally too far.^8^ The demerit of sharp uterine incision is increased blood loss from incision line and trauma to fetus. The controlled expansion thus protecting uterine artery and parametrical veins is the main advantage of sharp uterine incision.^9^

There are two variants of blunt uterine incision either up and down traction or by transverse lateral traction. Cephalad caudad traction offers some defense against unrestrained extension into broad ligament and reductions in tissue damage. Previous studies conducted locally and internationally have showed a significant difference in mean hemoglobin (Hb) and hematocrit (Hct) fall before surgery and 48 hrs. later between two groups.^6^ local studies done previously, have been conducted on woman with primary and repeat, emergency or elective cesarean section and also on woman with arrest disorders creating a bias.^7^ So our study aimed to compare the role of blunt versus sharp uterine incision on maternal blood loss estimated with fall of HB and HCT in primary elective cesarean section, thus excluding risk factors for excessive bleeding such as emergency cesarean section, repeat cesarean section and arrest disorders. Study would help to set a protocol for type of maternal uterine incision which will reduce maternal blood loss (fall in HB and HCT) at cesarean section thereby reducing maternal morbidity.

## METHODS

The trial was conducted at the Department of Obstetrics and Gynecology Pakistan Ordinance Factory Hospital, Wah Cantt from 13^th^ January 2012 till 14^th^ July 2012. Ethical committee approval was taken dated 13^th^ January, 2012; Ref No. 1657 /REU/OBG-2010-153-4640. While trial registration was done dated 9 January, 2021 (Randomized Control Trial ID AEARCTR-0006916). Sample size was calculated by WHO sample size calculator by taking confidence interval=95%, power of testing=80%, pooled standard deviation=2.6, test value of population means= 2.4, anticipated population mean =4.6. Group-A included 40 patients with blunt uterine incision while Group-B included 40 patients with sharp uterine incision. Non probability consecutive sampling had been used for sample selection. All women with Para 4 and less undergoing primary, elective lower segment cesarean section with placenta situated in upper segment were included in the study. Women with history of coagulation defects, decreased hemoglobin due to any cause, with past history of chronic medical diseases and a history of thrombophilia’s, multiple gestations, patients with large for dates pregnancy, patients with severe hypertensive disorders, and patients operated in emergency for antepartum hemorrhage and arrest disorders were excluded from the study.

Patients who fulfilled the inclusion criteria were admitted through OPD after taking history, examination and investigations. Informed consent was taken for scheduled elective lower segment cesarean section. Patients were allocated to either Group-By randomization through consecutive sampling technique. Second year postgraduate trainee performed the procedure under supervision of consultant. Prophylactic antibiotic was given before Pfannenstiel incision to all women. After skin incision with scalpel, subcutaneous tissue was opened with scalpel as little as possible and extended by blunt dissection. A small transverse niche 1-2cm length was given at uterus in the lower segment with scalpel and expanded bluntly by pulling fingers apart laterally and upwards in Group-A. Sharp expansion of small incision on uterus was done by cutting laterally with bandage scissor in Group-B. Placenta and membranes were removed by controlled cord traction method. Uterine incision was stitched in double layer with vicryl. Visceral and parietal peritoneum was not closed. Rectus sheath was closed and for skin subcuticular closure technique were used. peri operative blood loss was measured by comparing immediate preoperative Hct and Hb with Hct and Hb tested 48 hr after the operation. All patients were reviewed by trainee researcher in postoperative period and findings were recorded in Performa. All data it was entered and analyzed using SPSS version 10. Mean and standard deviation is calculated for quantitative variables (age, gestational age, mean hematocrit and hemoglobin fall). Independent sample t-test is used for comparison of quantitative variables like (mean Hb and Hct) fall by both procedures. P value <0.05 it was considered statistically significant.

## RESULTS

The two groups were comparable in terms of age, parity and gestational age and indication of cesarean section (Table-I). Main outcome measures were the mean perioperative hematocrit and Hb fall among two groups. The mean fall in haemocrit and Hb was statistically significant in the participants of Group-B (sharp uterine incision) as compared to Group-A (blunt uterine incision). Table-II and III.

**Table I:** Demographic Profile(n=80).

*Demographic profile*	*Blunt uterine incision (mean ±SD)*	*Sharp*	*uterine incision (mean ±SD)*	*p-value*
Age (years)	29.25±2.8		28.32±2.2	P=0.11
Parity	0.78±1.21		0.50±.85	P=0.24
Gestational age (weeks)	38.76±0.6		38.27±1.78	P=0.104

**Table II:** Comparison of pre and postoperative hematocrit fall between 2 groups.

*Groups*	*No. of patients*	*Preoperative hematocrit (mean±SD)*	*Post-operative hematocrit (mean±SD)*	*mean HCT fall*
A(Blunt uterine incision)	40	37.841.26	34.61±1.9	3.21±1.3
B(Sharp uterine incision)	40	38.07±2.3	33.61±2.6	4.21±2.17
P-value		0.59	0.09	0.015

**Table III:** Comparison of pre- and post-operative Hb drop between 2 groups.

*Study group*	*No. of patients (N)*	*Preoperative Hb (mean±SD)*	*Postoperative Hb (mean±SD)*	*mean Hb fall*
A(Blunt uterine incision)	40	11.8±0.47	10.36±1.0	1.47±1.08
B (Sharp uterine incision)	40	12.02±0.50	10.05±0.79	1.95±0.85
P-value		0.14	0.08	0.031

## DISCUSSION

Cesarean section is one of the oldest procedures. Several surgical techniques have been developed over passage of time like vertical and transverse incision in uterine wall but the most popular is the transverse uterine incision and is used commonly by obstetricians.^10^,^11^ This study was performed to compare the intra-operative blood loss during casraean section between sharp and blunt uterine incision.

For drawing a valid conclusion, it was really necessary to rule out other risk factors for operative blood loss during a cesarean section. we used strict criteria in choosing patients for this study. Several factors are reported to increase the blood loss such as advanced maternal age, multi-fetal gestations, prolonged labor, multiparity, previous Cesarean delivery, Placenta previa, and placenta accreta, chorioamnionitis and general anesthesia.^12^

We selected woman with single term pregnancy with parity less than four, no previous cesarean sections and placenta located in upper segment. Patients were selected according to WHO criteria in terms of Hb of >11.5 g/dl. The difference between two groups was also not significant (p-value =0.14). The mean age of mother was also 28.3 years and change were not significant among two groups (p-value=0.11). The type of anesthesia selected was spinal with standard protocol and didn’t use general anesthesia as it is associated with increased intraoperative blood loss. Emergency cesarean delivery is also associated with increased blood loss as compared with elective cesarean section, it is not possible to take an informed consent regarding type of uterine incision during most of emergency procedure so elective cesarean sections were only chosen.

Another important factor which produced biased in previous studies were arrest disorders because they are known to be associated with increased unintended extensions^13^ and increased blood loss so they were also excluded from study.

There are different methods for estimation of blood loss such as gravimetric method, laboratory methods (Hct fall and Hb fall). All of them are comparable but in gravitational method there is slightly more chances of error of estimation of blood loss(25%).^7^ So the laboratory methods are used in this study because of error to be produced in estimation of blood loss and surgeon may be aware of type of technique that is used and this could affect the estimation of blood loss but this could not affect change in Hct and Hb so Hct and Hb is used for estimation of blood loss and 2^nd^ reading is taken 48 hour after the operation.

Our results showed post operative hematocrit fall of (mean3.21±SD1.30) in blunt type of uterine incision versus (mean 4.2±SD2.17) in sharp uterine incision the difference among two is statistically significant markedly (p value =0.015) and it is analogous to the results of Sekhavat et al (2009) which also shows a statistically significant Hct fall in sharp Group-As compared to group of blunt uterine incision. Our study is also comparable with the Sekhavat et al in terms of selection of patient, however only thing lacking in our study was estimation of blood volume (reason already discussed), and the need for blood transfusion.^6^ However, no blood transfusion is needed because of there is no fall of hematocrit >10% that is used as definition massive hemorrhage.^7^ Our results of post operative Hb drop were also comparable to Sekhavat et al.^6^ and they postulated that increased blood loss may be due to increased extension of uterine incision in sharp Group-As compared to blunt uterine incision. Another study done by Magnn EF (2002) at university of Missippi Medical Centre concluded that sharply expanding the uterine incision during cesarean delivery is associated with significantly increased risk of intraoperative blood loss and need for blood transfusion. They came to conclusion that increased blood loss during sharp expansion of uterine incision may be due to increased blood loss from incised edge itself and also because of more extension of sharp uterine incision as compared to blunt uterine incision and overall increase in number of extension had a significant impact on amount of blood loss in cesarean delivery.^14^ However, a recent systemic review with meta-analysis by Xu LL (2012) also reviewed multiple trials on blunt versus sharp uterine incision. The results after reviewing three trials of Sekhavat et al.^6^ and Magann EF et al.^14^ showed a trend towards blunt type of uterine incision which is related with less blood.^15^ According to analysis blood loss was more with sharp uterine incision. The results reached the level of statistical in terms of blood loss estimation (vol) but not in terms of Hb and Hct drop. A Cochrane review by Dodd JM et al. (2008) on surgical techniques for uterine incision showed that blunt dissection of uterine incision was associated with a reduction in mean blood loss at time of procedure when compared with sharp dissection of uterine incision. (MD-43,95%CI- 66.12 to-19.88) and no statistical significance in terms of blood transfusion.^9^

Another study conducted locally by Shamsi et al.^7^ (2005) Postulated that both types of uterine incision are comparable in terms of blood loss during cesarean section because blunt type of uterine incision is associated with more extension of uterine incision as compared to sharp uterine incision however difference among two is not statistically significant.^7^

Our study support the results of Xu LL et al. and postulate that blunt type of uterine incision is associated with less blood loss when the bias of arrest disorder is excluded because if myometrial planes are used bluntly for cutting they are associated with less oozing from myometrial edges and sharp expansion is associated with increased bleeding because of increased bleeding from the incised edges itself, from muscle ooze, traumatized vasculature or secondary to a greater forward extension of distal incision.^15^,^16^

Further studies are needed for evaluation of type of uterine incision in patient with repeat cesarean section as there is increased risk of inadvertent uterine extension when blunt uterine incision is used.^17^,^18^

To validate the superiority or otherwise of the blunt uterine incision for hysterotomy during a cesarean section larger multicentric prospective studies are needed aimed at assessing morbidity with particular reference to operative time, changes in hematocrit and blood loss and hemoglobin fall and extension of uterine incision with standardized protocols regarding indication for cesarean section and arrest disorders.^19^,^20^

### Limitations of the study

The principal limitation was time factor. As this study was being conducted as a fulfillment of training requirement of the examination of FCPS in Gynaecology/Obstetrics, its duration had to be contained to six months of training period. This leads to the emergence of few states of affairs which produced bias. First of these was small sample size. Each of these can have a bearing on the ultimate outcome of the point under consideration. Therefore, any of these could have affected our results in any direction unknowingly.

## CONCLUSION

Blunt expansion of uterine incision is better than the sharp uterine incision at cesarean section as it is associated with less hemoglobin and hematocrit fall.

### Authors’ Contribution:

**FAIZA:** Conceived, designed and did statistical analysis & editing of manuscript. Responsible for integrity and accuracy of work

**NRK:** Results and Discussion.

**BA:** Introduction and literature Review.

**FS:** Final approval of the manuscript.
